# Integrated Metabolomics and Proteomics Analysis of the Myocardium in a Mouse Model of Acute Viral Myocarditis

**DOI:** 10.1002/iid3.70151

**Published:** 2025-02-06

**Authors:** Yimin Xue, Jiuyun Zhang, Mingguang Chen, Qiaolian Fan, Tingfeng Huang, Jun Ke, Feng Chen

**Affiliations:** ^1^ Fourth Department of Critical Care Medicine Shengli Clinical Medical College of Fujian Medical University, Fujian Provincial Hospital, Fuzhou University Affiliated Provincial Hospital, Fujian Provincial Key Laboratory of Emergency Medicine Fuzhou Fujian China; ^2^ Department of Emergency Shengli Clinical Medical College of Fujian Medical University, Fujian Provincial Hospital, Fuzhou University Affiliated Provincial Hospital, Fujian Provincial Key Laboratory of Emergency Medicine Fuzhou Fujian China

**Keywords:** acute viral myocarditis, Coxsackievirus B3, metabolomics, myocardium, proteomics

## Abstract

**Background:**

Acute viral myocarditis (AVMC) is a common inflammatory disease affecting the myocardium and is often accompanied by severe metabolic disturbances. The molecular mechanisms underlying this disease are complex and not yet fully understood.

**Methods and Results:**

Coxsackievirus B3 (CVB3)‐induced AVMC mouse models were established. By integrating ultra‐high‐performance liquid chromatography‐tandem mass spectrometry (UHPLC‐MS/MS)‐based metabolomics and data‐independent acquisition (DIA)‐based proteomics, we aimed to investigate the global influence of CVB3 infection on the myocardial metabolome and proteome in mice. Based on the criterion of OPLS‐DA VIP > 1.0 and *p* value < 0.05, a total of 149 differential metabolites (DMs) were identified, including 64 upregulated and 85 downregulated metabolites. Bioinformatics analysis revealed that these DMs were mostly enriched in Global and overview maps (Metabolic pathways), Energy metabolism (Sulfur metabolism and Nitrogen metabolism), Amino acid metabolism (Taurine and hypotaurine metabolism, Lysine degradation, and Arginine and proline metabolism), and Carbohydrate metabolism (Propanoate metabolism). Differential analysis also identified 1385 differential proteins (DPs) between the two groups (|Fold Change| >1.5 and *p* value < 0.05), including 1092 upregulated and 293 downregulated proteins. Gene Ontology (GO) and Kyoto Encyclopedia of Genes and Genomes (KEGG) enrichment analyses of DPs indicated that metabolism‐related pathways were significant components of the AVMC process. Next, we mined many DPs engaged in the above metabolic pathways through an integrated analysis of KEGG pathway‐based metabolomics and proteomics data.

**Conclusions:**

Our integrated metabolomics and proteomics analysis revealed characteristic alterations in metabolites and proteins in the myocardium of AVMC, as well as the associations between them. This not only extends the existing understanding of the molecular basis of the pathogenesis and progression of AVMC but also suggests new directions for its treatment.

## Graphical abstract

In this article, Coxsackievirus B3 (CVB3)‐induced AVMC mouse models were established. By integrating ultra‐high‐performance liquid chromatography‐tandem mass spectrometry (UHPLC‐MS/MS)‐based metabolomics and data‐independent acquisition (DIA)‐based proteomics analyses, we aimed to investigate the global influence of CVB3 infection on the myocardial metabolome and proteome in mice.

## 1 Introduction

Acute viral myocarditis (AVMC) is an immune‐mediated acute inflammatory disease of the myocardium, mainly caused by cardiotropic virus infection. Coxsackievirus B3 (CVB3), an enterovirus of the *Picornaviridae* family, is considered the most common etiological agent [1]. CVB3 has been widely used in AVMC research since it was first employed to induce myocarditis in mice by Woodruff in 1974 [2]. CVB3 infection in mice closely mimics the clinical and pathological features of AVMC caused by enteroviruses in humans, making it an effective model for studying the mechanisms of AVMC and testing potential therapeutic interventions [2, 3]. Although most patients with AVMC have mild symptoms, a proportion of cases progress to fulminant myocarditis (FM), dilated cardiomyopathy (DCM), and even sudden death [4, 5]. Extensive studies suggest that the pathogenic mechanisms underlying AVMC primarily involve direct viral invasion, immune dysregulation, and diffuse myocardial injury and remodeling [3]. However, our advances in AVMC pathophysiology have not yet translated into improved clinical treatment options.

The heart is the most metabolically demanding organ in the human body, which must continuously produce large amounts of adenosine triphosphate (ATP) to sustain contractile function by metabolizing an array of fuels, such as fatty acids, glucose, lactate, pyruvate, and amino acids [6, 7]. Using high‐throughput RNA sequencing, we found that the most significant enrichment pathway for differentially expressed mRNAs in AVMC was the metabolic pathway [8]. Viral infection damages mitochondrial ultrastructure, causing metabolic disorders and metabolite accumulation in cardiomyocytes, ultimately leading to an imbalance in energy supply and demand that accelerates the deterioration of cardiac function [9]. Recently, several metabolites have been demonstrated to serve essential roles in AVMC development. For example, kynurenine 3‐monooxygenase deficiency reduces mortality in mice with AVMC by increasing serum kynurenine pathway metabolites and decreasing chemokine production [10]. Nano‐α‐linolenic acid exerts a protective effect against AVMC in a dose‐dependent manner [11]. The latest study by Zhou et al. [12] found that epoxyeicosatrienoic acids can prevent the progression of CVB3‐induced AVMC, particularly by increasing IFN production to promote viral resistance. Therefore, a full understanding of metabolite alterations in AVMC may help to uncover new therapeutic targets and provide new mechanisms for clinical intervention.

As a powerful analytical tool, metabolomics has been widely used to explore changes in the global cardiometabolic profile of individuals with cardiovascular diseases. Currently, the main metabolomics analysis platforms include ultra‐high‐performance liquid chromatography‐tandem mass spectrometry (UHPLC‐MS/MS), gas chromatography‐mass spectrometry (GC‐MS), and nuclear magnetic resonance (NMR), each with its own advantages and shortcomings [13]. Kong et al. [14] recently identified differential metabolites (DMs) in the myocardium of AVMC, chronic viral myocarditis (CVMC), and DCM mice using NMR‐based metabolomics. UHPLC‐MS/MS is the most commonly used platform for metabolic profiling due to its higher sensitivity and superior detection capability compared with NMR, mainly for thermally unstable, nonvolatile, and polar compounds, unlike GC‐MS [15, 16]. However, myocardial metabolomics studies based on UHPLC‐MS/MS in AVMC are still lacking. Proteomics, an important complement to metabolomics, represents the summative effects of gene function and can be used to identify differential proteins (DPs) that directly affect metabolic processes [17]. The integrated analysis of metabolomics and proteomics contributes to a more comprehensive systematic assessment of physiological states, especially for elucidating pathogenesis and identifying biomarkers through modern data analysis [18, 19].

To fill this gap, we established a CVB3‐induced AVMC mouse model. Subsequently, an untargeted UHPLC‐MS/MS‐based metabolomics approach and a data‐independent acquisition (DIA)‐based proteomics method were performed in the myocardium. Finally, we analyzed the Kyoto Encyclopedia of Genes and Genomes (KEGG) metabolic pathways shared by DMs and DPs. Our research will provide a deeper understanding of the pathophysiology underlying AVMC.

## 2 Materials and Methods

### 2.1 Animal Handling

All experimental protocols followed the Principles of Laboratory Animal Care (People's Republic of China) and were approved by the Institutional Animal Care and Use Committee of Fujian Medical University (License No. IACUC FJMU 2023‐0278). The research staff received special training in animal care and handling provided by Fujian Medical University. Thirty‐six specific pathogen‐free (SPF) male BALB/c mice aged 6 weeks were purchased from Beijing SiPeiFu Biotechnology Co. Ltd. [License No. SCXK (Jing) 2019‐0010]. The animals were housed in a temperature‐controlled environment at 24°C with 12‐h day‐night cycles and had free access to food and water. After a 3‐day adaptation period, the mice were assigned to the Control (*n* = 13) or AVMC group (*n* = 23) according to a random number table, and no blinding was performed. CVB3‐induced AVMC mouse models were constructed by intraperitoneal injection of CVB3 (Nancy strain; 2 × 10^5^ PFU per mouse), while the Control mice were injected with an equal volume of phosphate‐buffered saline (PBS). The overall experimental period lasted 7 days (from Day 0 to Day 7). Daily observations were performed to evaluate the external signs and behavioral activities of the mice. Mice that died during the experiment were excluded from further analysis. After 7 days, the surviving mice were euthanized via cervical dislocation under isoflurane (2%) anesthesia. Their hearts were collected for histological examination, untargeted metabolomics, and DIA proteomics. All efforts were made to minimize the suffering of the mice.

### 2.2 Hematoxylin and Eosin (HE) Staining

Five mice were randomly selected from each group for cardiac histology. After examining the gross appearance, the heart was cut transversely. HE staining was conducted according to routine protocols. In brief, the collected heart tissues were fixed, dehydrated, and embedded in paraffin. The tissue blocks were then cut into 5‐μm sections and stained with HE. The sections were observed and scanned using a Motic EasyScan Digital Slide Scanner, and the severity of inflammation was scored using the method described previously [20].

### 2.3 Metabolite Extraction and UHPLC‐MS/MS Analysis

The hearts of the remaining mice in each group were horizontally divided into two portions: one for untargeted metabolomics and the other for proteomics. For heart metabolite extraction, tissue samples were ground in liquid nitrogen, and the homogenates were resuspended in 500 μL of prechilled 80% methanol and vortexed for 4 min. The suspensions were kept on ice for 5 min and centrifuged at 15,000*g* for 20 min at 4°C. Some supernatants were diluted with MS‐grade water to a final concentration of 53% methanol. Subsequently, the samples were centrifuged at 15,000*g* for 20 min at 4°C, and the supernatants were applied to UHPLC‐MS/MS analysis [21].

Analyses were performed using a Vanquish UHPLC system coupled with an Orbitrap Q Exactive HF‐X mass spectrometer (Thermo Fisher Scientific). The detailed experimental conditions for UHPLC‐MS/MS are described in Supporting Information S2: Table S1. For quality control (QC) and preprocessing, a pooled sample was prepared to assess the analytical variability by mixing equal volumes (20 μL) of the supernatant from each sample.

### 2.4 Data Processing, DMs Identification, and Functional Prediction

The raw data files generated by UHPLC‐MS/MS were processed using Compound Discoverer v3.1 software (Thermo Fisher Scientific) for peak alignment, peak selection, and quantification of each metabolite. The main parameter settings were the same as those described previously [22]. Then, the peak area was quantified, the target ions were integrated, and the molecular formula was predicted based on the molecular ion peak and fragment ions. This information was then compared with the MassList, mzCloud, and mzVault databases to identify the metabolites. The background ions were removed with blank samples (53% methanol solution containing 0.1% formic acid), and the quantitative results were normalized with QC samples. Statistical analyses were performed using the statistical software R (v3.4.3), Python (v2.7.6), and CentOS (v6.6).

Principal component analysis (PCA) and orthogonal projection to latent structures‐discriminant analysis (OPLS‐DA) were performed to visualize changes in metabolic profiles between the Control and AVMC groups [23]. A 200‐cycle permutation test was conducted to assess the robustness and predictive ability of the OPLS‐DA model. The variable importance in the projection (VIP) value of each variable in the OPLS‐DA model was calculated to indicate its contribution to the classification. DMs were screened between two groups using the *t* test as a univariate analysis, and those with VIP > 1.0 and *p* value < 0.05 were considered differentially expressed. Meanwhile, the volcano plot and heatmap were created to visualize the DMs by the R package. Finally, these DMs were annotated with the KEGG compound database (http://www.kegg.jp/kegg/compound/) and the Human Metabolome Database (HMDB, http://www.hmdb.ca/), and then mapped to the KEGG pathway database (http://www.kegg.jp/kegg/pathway.html). The metabolic pathway analysis was also performed using the MetPA tool in MetaboAnalyst 5.0 (http://www.metaboanalyst.ca/MetaboAnalyst/) [24].

### 2.5 Protein Extraction, Preparation, and Nano‐HPLC‐MS/MS Analysis

Myocardial tissue samples were suspended in protein lysis buffer (1% SDS, 8 M urea), which included appropriate protease inhibitors to inhibit protease activity, and then the mixture was vortexed to mix well and processed twice through a high‐throughput tissue grinder. After precipitation for 30 min at 4°C (vortexing every 10 min), the sample was centrifuged at 15,000*g* for 20 min at 4°C. The supernatant was collected, and the protein concentration was determined by the bicinchoninic acid (BCA) method. Finally, equal amounts of protein (15 μg/lane) were subjected to 12% SDS‐PAGE gel electrophoresis. The gels were stained with Coomassie Brilliant Blue R‐250 and decolored until the bands were clearly visible.

Sample preparation included the processes of protein denaturation, reduction, alkylation, trypsin digestion, and peptide cleanup, according to the protocol provided in the iST Sample Preparation kit (PreOmics). Briefly, 50 µL of lysis buffer was added and heated at 95°C, 200*g* with stirring for 10 min. After cooling to room temperature (RT), trypsin digestion buffer was added, and the mixture was incubated at 37°C, 100*g* with shaking for 2 h. Subsequently, the samples were cleaned and desalted, and the peptides were eluted with elution buffer (2 × 100 µL) and lyophilized using SpeedVac.

A high pH reversed‐phase chromatography step was used to separate the complex mixture of peptides before nano‐HPLC‐MS/MS analysis. The mixed peptide samples were resuspended in buffer A (20 mM ammonium formate, pH 10.0, adjusted with ammonium hydroxide), loaded onto a reverse‐phase column (XBridge C18 column, 4.6 × 250 mm, 5 μm, Waters Corporation) using an Ultimate 3000 system (Thermo Fisher Scientific), separated, and eluted using a linear gradient of 5% to 45% buffer B (20 mM ammonium formate in 80% ACN, pH 10.0, adjusted with ammonium hydroxide) for 40 min. The column flow rate was maintained at 1 mL/min, and the column temperature was maintained at 30°C. The collected fractions were then concatenated into 10 fractions and dried in a vacuum centrifuge.

The peptides were redissolved in 30 μL solvent A (0.1% formic acid aqueous solution) and analyzed by on‐line nanospray LC‐MS/MS on an Orbitrap Fusion Lumos mass spectrometer coupled to an EASY‐nLC 1200 system (Thermo Fisher Scientific). Three microliters of the sample was separated on the analytical column (Acclaim PepMap C18, 75 μm × 25 cm) using a 120‐min gradient, from 5% to 35% in solvent B (0.1% formic acid in ACN). The column flow rate was maintained at 200 nL/min with a column temperature of 40°C, and the electrospray voltage was set to 2 kV. The mass spectrometer automatically switched between MS and MS/MS modes under the DIA configuration, and the detailed parameter settings are shown in Supporting Information S2: Table S2.

### 2.6 Data Processing, DPs Identification, and Bioinformatics Analysis

The DIA raw data were analyzed using Spectronaut 18 (Biognosys AG) with the default settings, and the ideal extraction window was dynamically determined based on the iRT calibration and gradient stability. A *Q*‐value (FDR) cutoff on the precursor and protein level was applied at 1%. Decoy generation was set to apply a random number of amino acid position swamps (min = 2, max = length/2). All selected precursors passing the filters were used for MS1 quantification. Proteins were quantified using the average of the top three peptide MS1 areas, yielding raw protein abundances. The thresholds of |Fold Change | > 1.5 and *p* value < 0.05 were used to identify DPs.

The DPs were further assigned to the Gene Ontology (GO) database (http://www.geneontology.org/), where the proteins were divided into three main categories: biological process (BP), molecular function (MF), and cellular component (CC). Pathway enrichment analysis was conducted using the KEGG database. Differences were considered to be statistically significant at a *p* value < 0.05.

### 2.7 Integrated Analysis of Metabolomics and Proteomics

All DMs and DPs were queried and mapped to KEGG‐based pathways. R version 3.4.1 was used to combine KEGG annotation and enrichment results of metabolomics and proteomics. Venn diagrams and bar graphs were plotted to combine the results of the two omics approaches.

### 2.8 Statistical Analysis

Data analysis was conducted using GraphPad Prism software (v8.0.1, GraphPad Software Inc.). The Mann–Whitney *U* test was used to evaluate differences in cardiac pathology scores between the two groups. A *p* value less than 0.05 was considered statistically significant.

## 3 Results

### 3.1 CVB3‐Induced AVMC in Mice

**Figure 1:**
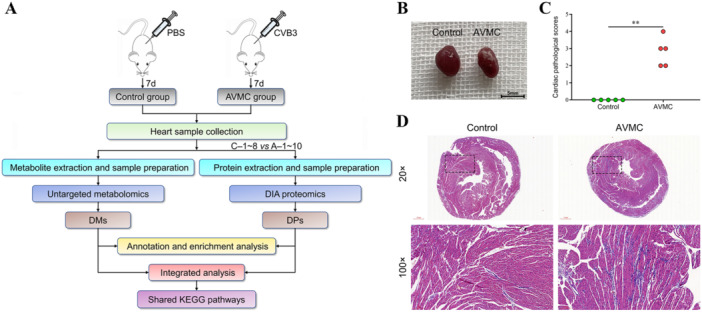
Overall experimental procedures and assessment of myocardial inflammation. (A) Schematic illustration showing the design of animal experiments. (B) Gross examination of the hearts from Control and AVMC mice on Day 7 postinfection. (C) Comparison of cardiac pathological scores between the two groups. Each dot represents an individual mouse. (D) Representative HE staining images of heart sections from the two groups (scale bar = 500 μm, 100 μm). **p value < 0.01.

The animal grouping and experimental design are shown in Figure 1A. During the experiment, the mice in the Control group were in good condition, exhibiting shiny fur, increased weight, a normal diet, and flexible responses. In contrast, the AVMC mice exhibited obvious viral infection symptoms, such as weakness, irritability, lusterless hair, anorexia, and weight loss, and eight of them died before the end of the experiment. Upon examining the gross appearance of the mouse hearts on Day 7 postinfection, we noted reduced heart size and apparent cellulose‐like exudation on the epicardial surface of the AVMC mice (Figure 1B). To evaluate the severity of AVMC in more detail, HE staining and histological analysis were performed on transverse sections of the hearts (Figure 1C,D). The AVMC group displayed significant focal necrosis, inflammatory cell infiltration, myocardial fiber collapse, and higher cardiac pathological scores, while no obvious histological abnormalities were observed in the Control group. These findings suggest that the CVB3‐induced AVMC mouse models were successfully established.

### 3.2 Altered Myocardial Metabolomic Profiles in AVMC Mice

**Figure 2:**
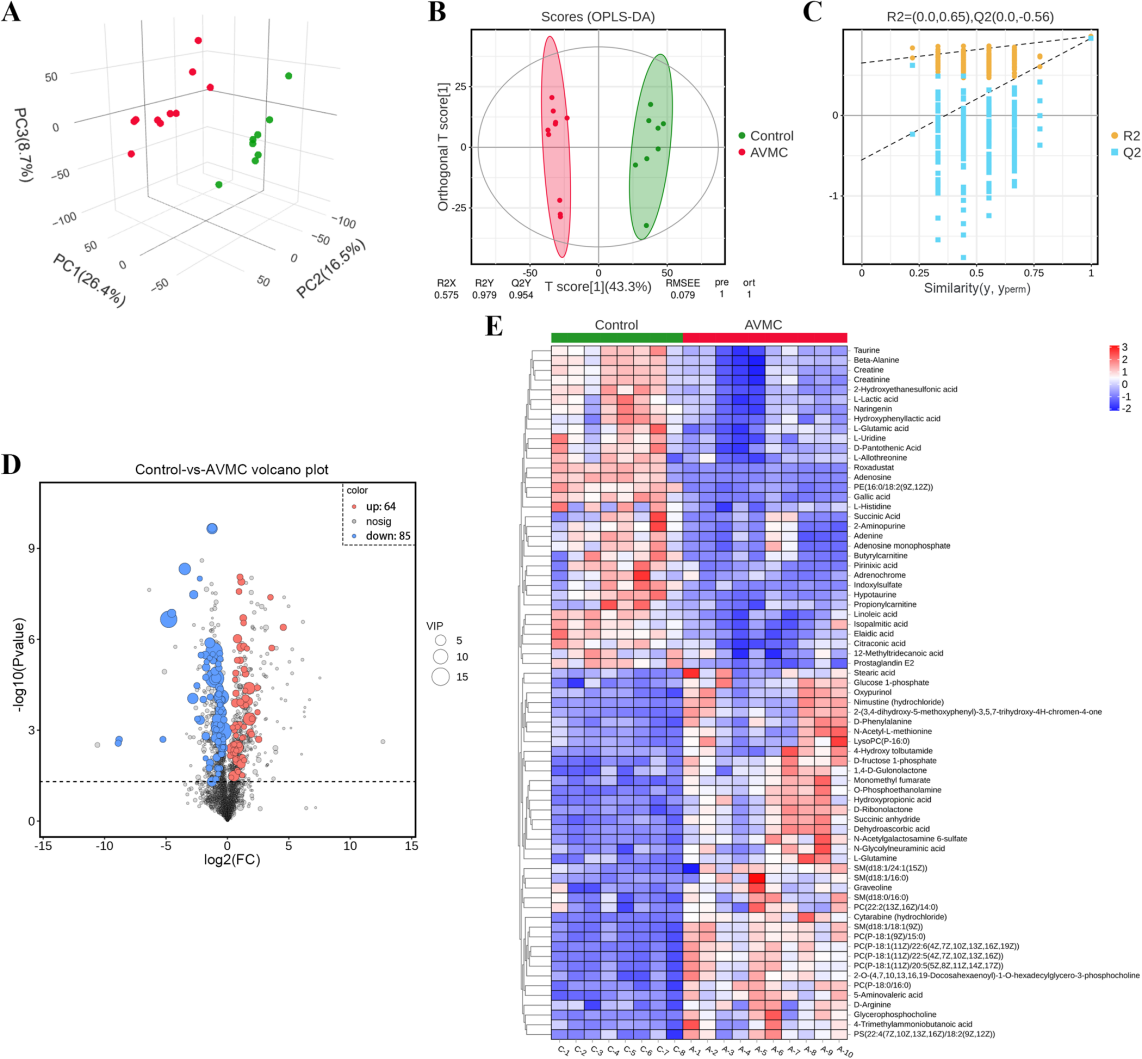
Altered myocardial metabolomic profiles in AVMC mice. (A) 3D scatter plot of PCA results. (B) OPLS‐DA analysis showing good discrimination between the Control and AVMC groups (R2X = 0.575, R2Y = 0.979, Q2Y = 0.954). (C) The permutation test (*n* = 200) for the OPLS‐DA model. (D) Volcano plot of DMs between the two groups. DMs were screened based on the criterion of VIP > 1.0 and *p* value < 0.05. Blue dots indicate downregulated metabolites, and red dots indicate upregulated metabolites. (E) Hierarchical clustering heat map analysis of DMs at the MS2 level between the two groups.

Subsequently, we applied an untargeted UHPLC‐MS/MS‐based metabolomics approach to determine the metabolome alterations between the Control (C‐1 ~ 8) and AVMC (A‐1 ~ 10) groups. After data processing and filtering, a total of 2671 metabolites were obtained, of which 2117 and 554 metabolites were annotated according to MS1 and MS2, respectively. A correlation heat map was used to show the relationship between samples, indicating that samples within the same group were highly correlated (Supporting Information S1: Figure S1). The 3D PCA plot demonstrated significant separation of metabolites between the two groups, with 26.4%, 16.5%, and 8.7% variation attributed to the principal components PC1, PC2, and PC3, respectively (Figure 2A). Similarly, the OPLS‐DA model was constructed to screen the DMs, and a significant division was observed between the two groups (R2X = 0.575, R2Y = 0.979, Q2Y = 0.954; Figure 2B). Following the permutation test, the results indicated that the OPLS‐DA model was reliable without overfitting, as the *R*2 and *Q*2 values were lower than the original point (Figure 2C).

To screen the DMs, the VIP in the OPLS‐DA model (VIP > 1.0) and the *p* value from the Student's *t* test (*p* value < 0.05) were used as the criteria. Collectively, 149 DMs were identified between the two groups, including 64 upregulated and 85 downregulated metabolites (Supporting Information S2: Table S3 and Figure 2D). Of these, 7 metabolites in positive mode and 71 metabolites in negative mode were nonredundantly assigned at the MS1 level. Similarly, 28 metabolites in positive mode and 43 metabolites in negative mode, assigned at the MS2 level, were differentially expressed and displayed in a hierarchical clustering heatmap for better visualization (Figure 2E). Based on the HMDB classification, the top four superclasses to which the DMs belonged were Lipids and lipid‐like molecules (36.84%), Organic acids and derivatives (21.93%), Organoheterocyclic compounds (11.40%), and Organic oxygen compounds (8.77%) (Supporting Information S1: Figure S2). Additionally, Pearson correlation analysis of the top 40 DMs at the MS2 level revealed some correlation between different DMs (Supporting Information S1: Figure S3), suggesting that the identified DMs might cooperate with each other to participate in the pathogenesis of AVMC.

### 3.3 Pathways Associated With Myocardial DMs

**Figure 3:**
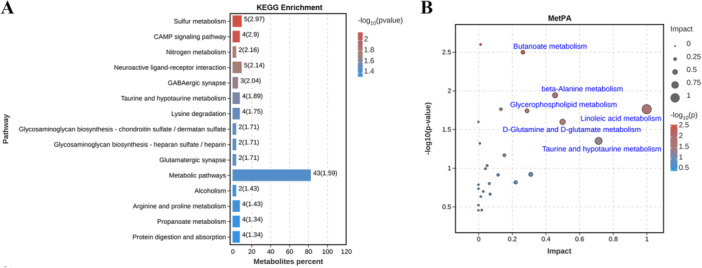
Pathways associated with myocardial DMs. (A) KEGG pathway enrichment analysis of DMs. The significantly enriched pathways are displayed by a bar plot (*p* value < 0.05), with the bar length indicating the number of enriched metabolites and the bar color indicating the ‐log_10_(*p* value). (B) Summary of pathway analysis using MetPA (top 25). The color and size of each circle are based on ‐log_10_(*p* value) and pathway impact value, respectively. Statistically significant pathways are annotated with their names in blue font (*p* value < 0.05 and impact value > 0.2).

Pathway analysis was conducted based on the KEGG database for the DMs. As shown in Supporting Information S2: Table S4 and Figure 3A, several key pathways were significantly enriched (*p* value < 0.05), such as Sulfur metabolism, Nitrogen metabolism, Taurine and hypotaurine metabolism, Lysine degradation, Metabolic pathways, Arginine and proline metabolism, and Propanoate metabolism. To identify significantly altered metabolic pathways in AVMC mice, we also performed metabolic pathway analysis using the MetaboAnalyst 5.0 webserver. A total of 51 metabolic pathways were identified, of which six pathways were significant (*p* value < 0.05 and impact value > 0.2), including Butanoate metabolism, β‐Alanine metabolism, Linoleic acid metabolism, Glycerophospholipid metabolism, D‐Glutamine and D‐glutamate metabolism, and Taurine and hypotaurine metabolism (Supporting Information S2: Table S5 and Figure 3B). These significantly altered metabolic pathways were related to Energy metabolism, Amino acid metabolism, and Carbohydrate metabolism, suggesting that CVB3 infection resulted in significant myocardial metabolic disturbances in mice.

### 3.4 Altered Myocardial Proteomics Profiles in AVMC Mice

**Figure 4:**
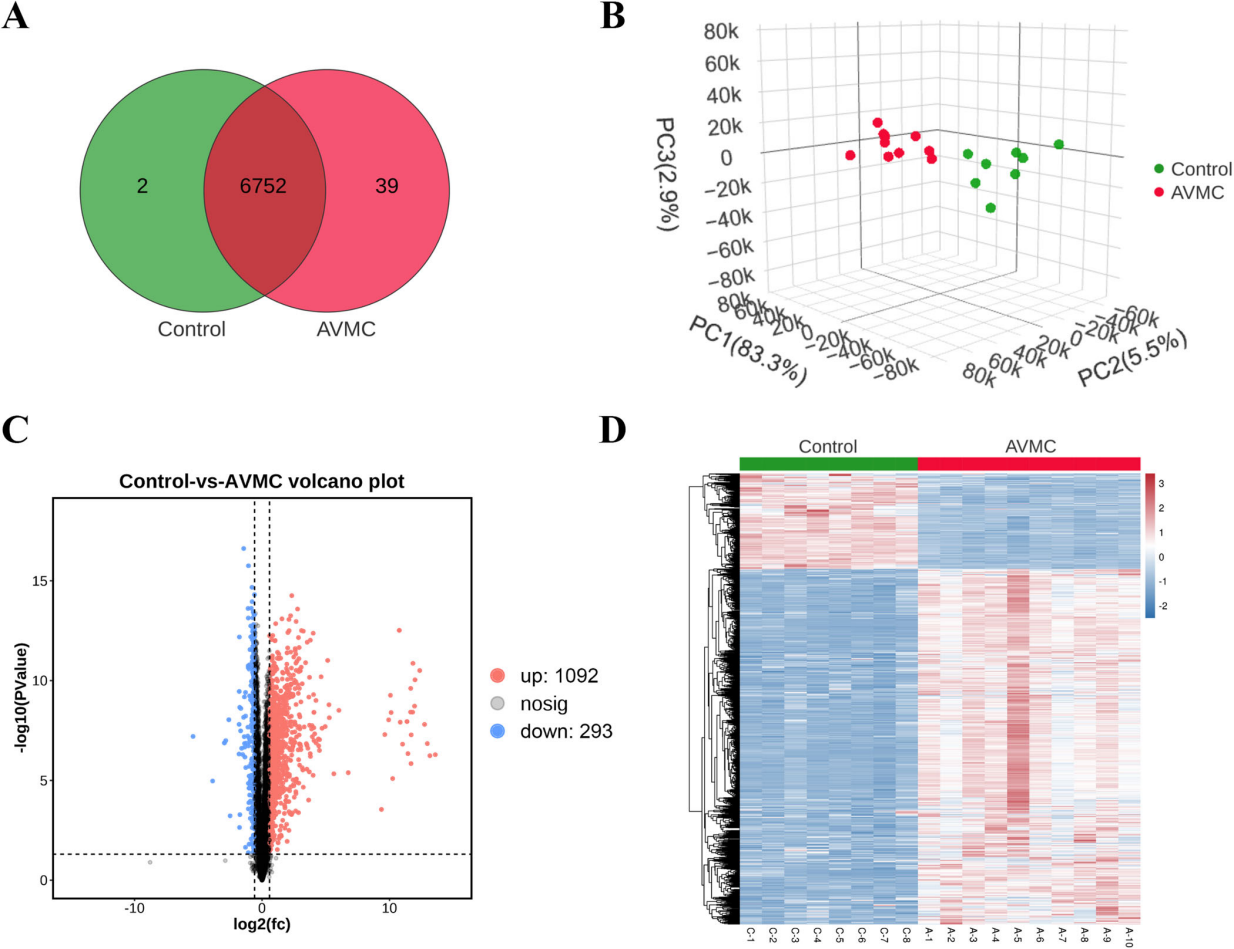
Altered myocardial proteomics profiles in AVMC mice. (A) Venn diagram of proteins identified in the Control and AVMC groups. (B) 3D PCA analysis of all samples based on their protein expression. (C) Volcano plot of DPs between the two groups. DPs were screened based on the criterion of |Fold Change| > 1.5 and *p* value < 0.05. Blue dots indicate downregulated proteins, and red dots indicate upregulated proteins. (D) Heatmap of DPs using unsupervised hierarchical clustering.

Myocardial tissue samples were also collected and sequenced using a DIA‐based proteomic method. A total of 6793 proteins were screened for further analysis, of which 6752 proteins were identified in both groups (Figure 4A). PCA and Pearson correlation analysis showed a clear distinction between the Control and AVMC groups, indicating significant differences in proteomic profiles between the two groups (Figure 4B and Supporting Information S1: Figure S4). DPs were then identified based on the criteria of |Fold Change| >1.5 and *p* value < 0.05. Volcano plot analysis showed that there were 1385 DPs (1092 upregulated and 293 downregulated) between the two groups (Supporting Information S2: Table S6 and Figure 4C). Meanwhile, DPs were ranked using unsupervised hierarchical clustering (Figure 4D), indicating the rationality and credibility of the AVMC mouse model for investigating the DPs between the two groups.

### 3.5 GO and KEGG Enrichment Analyses of DPs

**Figure 5:**
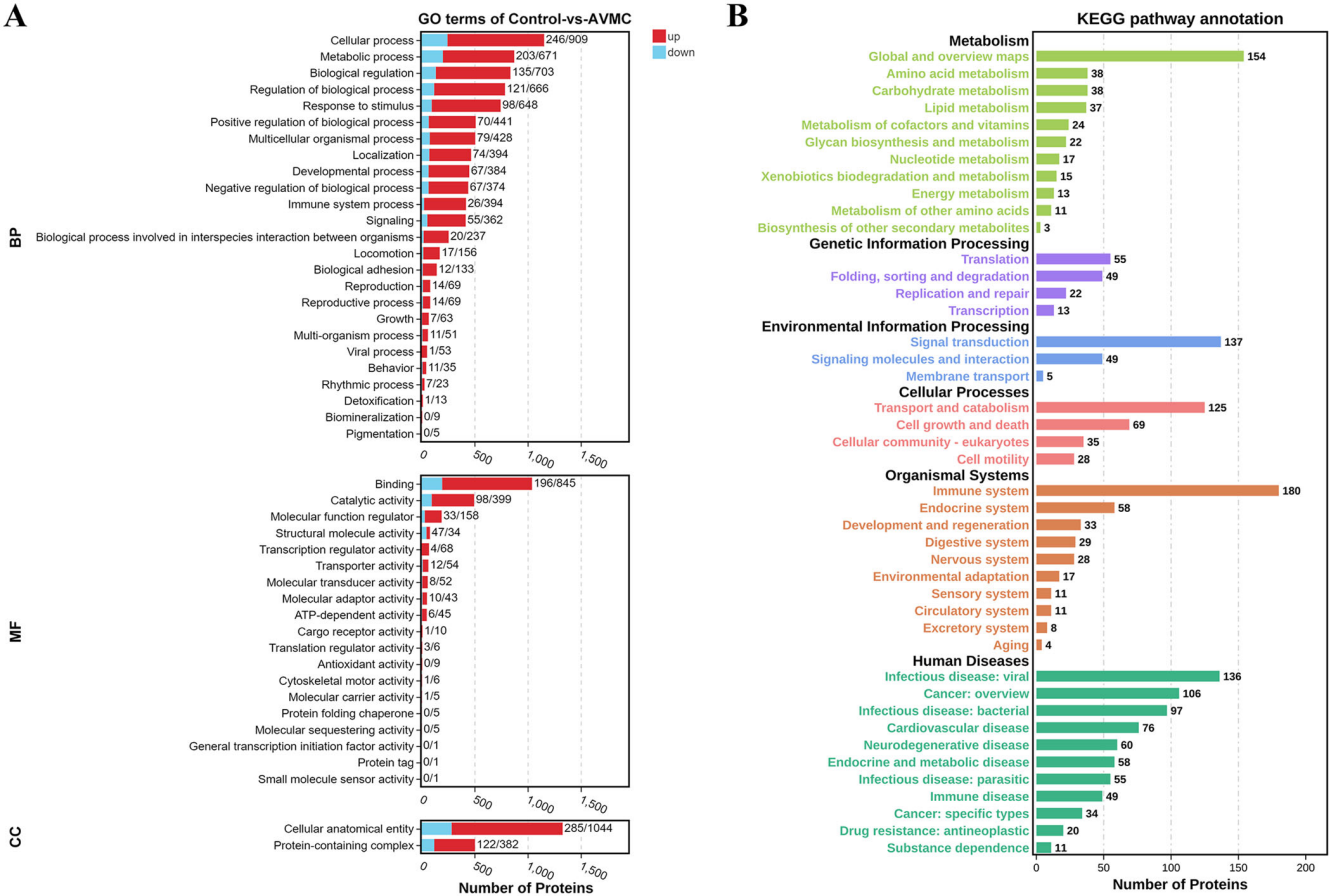
GO and KEGG enrichment analyses of DPs. (A) GO enrichment terms for the upregulated and downregulated DPs were grouped into three main ontologies: BP, CC, and MF. (B) KEGG pathway functional classification and annotation of all DPs. The number shown on the right side of each bar represents the number of proteins.

Next, GO and KEGG enrichment analyses were performed to determine the biological functions of the identified DPs. The significantly enriched GO terms for DPs are shown in Supporting Information S2: Table S7 and Figure 5A. Specifically, these DPs were enriched to a total of 46 GO terms and classified into categories of BP with 25 GO terms, MF with 19 GO terms, and CC with 2 GO terms. The top three enriched terms in the BP category were Cellular process, Metabolic process, and Biological regulation, with the number of DPs being 1155, 874, and 838, respectively. For the MF category, these DPs were mainly enriched in terms related to Binding, Catalytic activity, and Molecular function regulator. In the CC category, Cellular anatomical entity and Protein‐containing complex were the only two significantly enriched terms. To investigate the involved pathway of the DPs, we performed KEGG pathway annotation. As shown in Supporting Information S2: Table S8 and Figure 5B, metabolism‐related pathways, including Global and overview maps, Amino acid metabolism, Carbohydrate metabolism, Lipid metabolism, Metabolism of cofactors and vitamins, Glycan biosynthesis and metabolism, Nucleotide metabolism, Xenobiotics biodegradation and metabolism, Energy metabolism, Metabolism of other amino acids, and Biosynthesis of other secondary metabolites, were important components involved in the AVMC process. Thus, metabolic disturbances in AVMC might be accompanied by differential expression of many proteins.

### 3.6 Integrated Metabolomics and Proteomics Pathway Analysis

**Figure 6:**
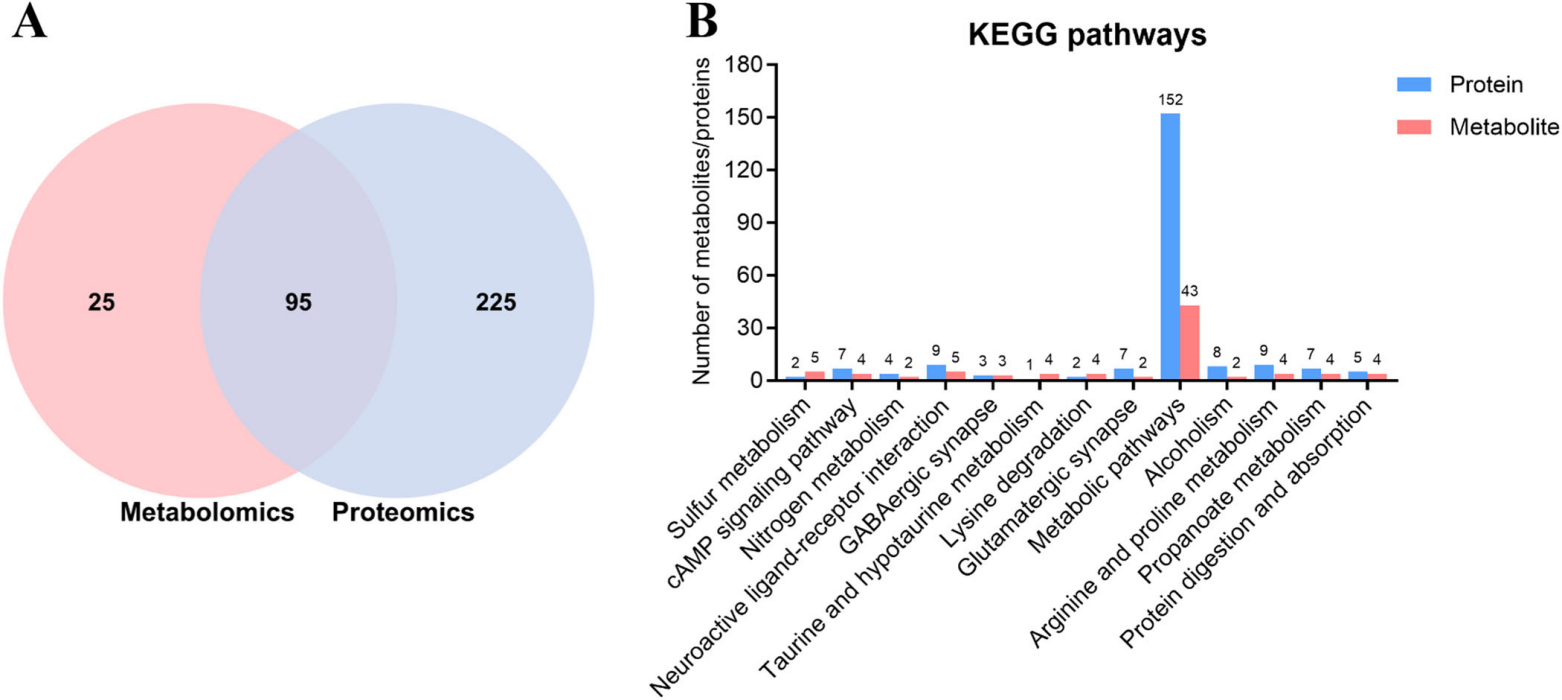
Integrated metabolomics and proteomics pathway analysis. (A) Venn diagram of the pathways in which DMs and DPs were involved. (B) The most enriched shared KEGG pathways were shown on the graph based on the *p* value of DMs < 0.05.

To associate the results of our metabolomics and proteomics analyses, we chose KEGG pathways as the carrier and conducted a mapping analysis based on the DMs and DPs. As shown in Supporting Information S2: Table S9 and Figure 6A, the Venn diagram revealed 95 KEGG pathways in which both DMs and DPs were involved, and a total of 54 metabolism‐related KEGG pathways were identified. Based on the *p* value of DMs less than 0.05, the most enriched shared KEGG pathways included Sulfur metabolism, cAMP signaling pathway, Nitrogen metabolism, Neuroactive ligand–receptor interaction, GABAergic synapse, Taurine and hypotaurine metabolism, Lysine degradation, Glutamatergic synapse, Metabolic pathways, Alcoholism, Arginine and proline metabolism, Propanoate metabolism, and Protein digestion and absorption (Figure 6B). Metabolic pathways contained the largest number of DMs and DPs.

## 4 Discussion

Myocardial metabolic disturbances have been shown to be strongly associated with the development of myocarditis [25]. In this study, we successfully constructed a CVB3‐induced AVMC mouse model and performed myocardial untargeted metabolomics analysis using UHPLC‐MS/MS. The results showed distinctly altered metabolic profiles and significantly disturbed metabolic pathways in the myocardium of AVMC mice, particularly Global and overview maps, Energy metabolism, Amino acid metabolism, and Carbohydrate metabolism. By integrating the proteomics data, we further mined many DPs that may be involved in the regulation of these metabolic pathways. To the best of our knowledge, this is the first study to reveal the association between the myocardial metabolome and proteome in AVMC mice. Our study provides a new perspective for elucidating the molecular mechanisms of AVMC.

Metabolites are the final products of all cellular activity and are closely related to phenotypes [26]. As the terminal of biological information transmission, they can reflect the physiological and pathological states of the heart. In this study, 149 DMs were identified in the myocardium of AVMC mice, with Lipid and lipid‐like molecules accounting for the largest proportion. This is in contrast to the study by Kong et al. [14], who identified a smaller number of DMs and mainly categorized them as Organic acids and derivatives, which may be related to the different detection methods and time points. Notably, 7 of the top 10 DMs with the most significant VIP values were markedly downregulated. For example, L‐Lactic acid with the highest VIP value is the end product of glycolysis generated from the reduction of pyruvate by lactate dehydrogenase [27]. Previous studies have shown that L‐Lactic acid is not only an important energy source but also serves as a multifunctional signaling molecule involved in a variety of physiological and pathological processes, including angiogenesis, neoplasia, inflammation, and immune regulation [28, 29, 30]. Interestingly, L‐Lactic acid can act as a natural suppressor of RIG‐I‐like receptor signaling by targeting mitochondrial antiviral signaling [31]. L‐Lactic acid reduction heightens type I IFN production to protect mice from viral infection, suggesting an antagonistic effect of glycolysis on antiviral immunity [31, 32]. Linoleic acid, the most abundant fatty acid found in cardiac mitochondria, is essential for the maintenance of mitochondrial function [33]. Mitochondrial damage caused by CVB3 infection is a key driver of cardiomyocyte death during AVMC progression and perhaps a major reason for Linoleic acid downregulation [9]. The cardioprotective effects of Linoleic acid intake have been demonstrated, and it may be a therapeutic agent for AVMC treatment in the future [34]. Adenosine, the most downregulated metabolite at the MS2 level, is a naturally occurring breakdown product of ATP and exerts multiple physiological effects, such as regulation of blood flow, heart rate, and cardiac contractility [35]. Adenosine preconditioning has been shown to protect the heart from ischemia‐reperfusion injury through diverse mechanisms, including increased antioxidant enzyme production, decreased inflammation, interaction with opioid receptors, and activation of various kinases (e.g., PKC, MAPK, Akt, and tyrosine kinase) [36]. Adenosine also inhibits IL‐2 production, thereby reducing CD4+ T cell activation and proliferation [37]. Thus, reduced levels of Adenosine in AVMC may lead to uncontrolled activation of lymphocytes and trigger persistent myocardial inflammation. These DMs provide fertile avenues for future mechanistic studies to identify novel targets for AVMC therapy.

Global and overview maps (Metabolic pathways) was the most abundant pathway for DMs enrichment with 152 DPs, implying a complex metabolic regulatory network during AVMC. Cardiac contractile performance is tightly coupled to Energy metabolism. CVB3 infection impairs energy homeostasis in cardiomyocytes, leading to decreased ATP production, increased reactive oxygen species (ROS), heightened glycolysis, and disturbances in amino acid and lipid metabolism, which in turn exacerbate inflammation and cell death [38]. In this study, Energy metabolism was one of the most disturbed metabolic pathways, including Sulfur metabolism and Nitrogen metabolism. Hydrogen sulfide (H_2_S) is an important compound produced during Sulfur metabolism. Tst and Ethe1, key enzymes involved in the mitochondrial oxidative catabolism of H_2_S [39, 40], were significantly downregulated in the Sulfur metabolism pathway. It has been shown that they oxidize H_2_S to sulfite in mitochondria for detoxification and possibly for the production of extra ATP [41]. Combined with metabonomic pathway enrichment analysis, it was also found that the metabolic pathway associated with L‐Glutamic acid and L‐Glutamine was Nitrogen metabolism, the basis of Energy metabolism. DPs involved in the Nitrogen metabolism pathway were annotated as Carbonic anhydrase (Car), including Car1, 3, 13, and 14, suggesting a strong correlation between Nitrogen metabolism and Carbon metabolism in AVMC [42]. Targeted regulation of key enzymes involved in Sulfur and Nitrogen metabolism may help improve Energy metabolism in cardiomyocytes, thereby alleviating AVMC progression.

The massive death of cardiomyocytes during AVMC results in protein degradation, in which most of the amino acids are reused to synthesize new proteins for tissue repair, but some of them also act as metabolic substrates to provide energy [43]. Hence, disordered Amino acid metabolism is an important component of the AVMC process. In this study, the enrichment of DMs in Taurine and hypotaurine metabolism, Lysine degradation, and Arginine and proline metabolism was the most significant. Taurine is the major osmolyte in cardiomyocytes, and it mainly affects osmoregulation, bile acid conjugation, cell proliferation, viability, and prevention of oxidant‐induced tissue damage. Hypotaurine, a precursor of taurine synthesis, is another metabolite possessing antioxidant capacity. This metabolic pathway is essential for the cellular stress response as taurine and hypotaurine are responsible for protection during osmotic stress and oxidative stress [44]. Notably, Ggt5, a key protein involved in this pathway, was significantly highly expressed in AVMC. It has been shown that overexpression of Ggt5 disturbs glutathione homeostasis and affects heme oxygenase‐1 levels, leading to excessive oxidative stress [45]. Thus, disturbances in Taurine and hypotaurine metabolism may be associated with an imbalance in the intracellular oxidative status of CVB3‐infected cardiomyocytes. Lysine is an essential amino acid in humans that can be degraded if present in excess. Razquin et al. [46] reported an association between excessive lysine levels and a high risk of diabetes‐concomitant cardiovascular diseases. Lysine degradation is beneficial because of the production of acetyl‐CoA for the citric acid cycle, and lysine catabolites also contribute to the relief of osmotic stress [47]. Lysine degradation primarily occurs in the mitochondria. We found that the mitochondrial matrix enzyme Gcdh, which is involved in this metabolic process, was significantly downregulated in AVMC, suggesting that the Lysine degradation process may be suppressed by CVB3 infection [48]. Arginine and proline metabolism is one of the central pathways in the biosynthesis of amino acids. Among them, arginine is a precursor for the synthesis of many important biomolecules, including nitric oxide (NO), polyamines, creatine, agmatine, proline, and glutamate [49]. Arginine also acts as a key regulator of multiple BPs, including gene expression, signal transduction, inflammation, and immune response [49]. This metabolic pathway may be significantly weakened in AVMC, supported by decreased levels of Creatine, Creatinine, and L‐glutamic acid, as well as elevated levels of Arginases (Arg1 and Arg2) and Glycine aminotransferase (Gatm) [50]. However, the detailed mechanisms of action of Arginine and proline metabolism in AVMC remain unknown and require further elucidation.

In addition to fatty acids, Carbohydrate metabolism is another source of energy for the heart, accounting for about 10%–40% of the heart's total energy supply [51]. In response to various stresses, the heart shifts its fuel substrate preference from fatty acids to carbohydrates through a mechanism of altered cardiac metabolic gene expression [52]. KEGG signaling pathway analysis in the present study revealed that Propanoate metabolism, which is involved in Carbohydrate metabolism, was significantly impacted. Succinic acid is an important intermediate in Propanoate metabolism and produces ATP via the gluconeogenesis pathway [53]. It is also a crucial signal connecting Carbohydrate metabolism with other metabolic pathways [54, 55]. Therefore, decreased levels of succinic acid indicate reduced ATP biosynthesis. Additionally, most of the proteins involved in the regulation of Propanoate metabolism, including Bckdha, Mcee, Suclg1, Mmut, Aldh6a1, and Dbt, were significantly downregulated, further confirming the marked inhibition of this pathway by CVB3 infection.

Like other viruses, CVB3 manipulates lipids in host cells to facilitate viral replication [56]. A recent study has shown that obesity exacerbates CVB3 infection through lipid‐induced mitochondrial ROS generation, suggesting a strong link between CVB3 infection and Lipid metabolism [57]. Based on the MetPA pathway analysis, we found that, in addition to Linoleic acid metabolism, Glycerophospholipid metabolism was also significantly affected. Among these, the most notable change was the upregulation of Glycerophosphocholine, a major component of cell membranes. The increase in Glycerophosphocholine likely reflects the degradation of membranes and elevates the risk of cardiovascular diseases [58]. However, its role in AVMC remains unclear.

There are some limitations of this study worth noting. First, since the information revealed by metabolomics is much narrower than that of proteomics, the integrated analysis cannot cover every aspect of the BP. Second, the integrated metabolomics and proteomics analysis of myocardium at different time points after CVB3 infection was not investigated in this study, and multiomics studies in female AVMC mice are also a direction of interest. Third, quantitative validation (e.g., western blot analysis and targeted metabolomics) of the molecules identified in this study is necessary before they can be generalized to other studies. Finally, targeting the regulation of key DMs and DPs within specific metabolic pathways will aid in identifying potential therapeutic interventions. This can be achieved through methods such as gene editing (e.g., CRISPR‐Cas9), RNA interference (e.g., siRNA/shRNA), pharmacological agents, epigenetic regulation, transcription factor modulation, and alterations in the microenvironment. Future integrated multiomics analyses in patients with AVMC, combined with longitudinal or interventional studies, are warranted to strengthen and broaden our findings.

## 5 Conclusion

In summary, this study presents a comprehensive analysis of metabolic and proteomic profiles in AVMC mice. Our results suggest that CVB3‐induced AVMC is closely related to several metabolic pathways that are accompanied by DP expression. These data provide further insights into the pathogenesis of AVMC and may help identify potential targets for improved clinical treatment.

## Author Contributions

**Yimin Xue:** conceptualization, data curation, formal analysis, investigation, methodology, writing – original draft, writing – review and editing. **Jiuyun Zhang:** conceptualization, investigation, methodology, writing – original draft. **Mingguang Chen:** data curation, investigation. **Qiaolian Fan:** data curation, investigation. **Tingfeng Huang:** investigation, software. **Jun Ke:** resources, software, supervision**. Feng Chen:** conceptualization, funding acquisition, supervision, validation, writing – review and editing.

## Ethics Statement

All animal protocols were evaluated and approved by the Institutional Animal Care and Use Committee of Fujian Medical University (Permit No. IACUC FJMU 2023‐0278).

## Consent

The authors have nothing to report.

## Conflicts of Interest

The authors declare no conflicts of interest.

## Supporting information

Supporting information.

Supporting information.

## Data Availability

Data sets are available from the corresponding author upon reasonable request.
