# Supplementary material for: Integrated Metabolomics and Proteomics Analysis of the Myocardium in a Mouse Model of Acute Viral Myocarditis
Source: Immun Inflamm Dis. 2025 Feb 6;13(2):e70151. doi: 10.1002/iid3.70151 (PMC11800238; doi:10.1002/iid3.70151)
Supplement: Supplementary file 1 — Supporting information. [file IID3-13-e70151-s002.docx]

**
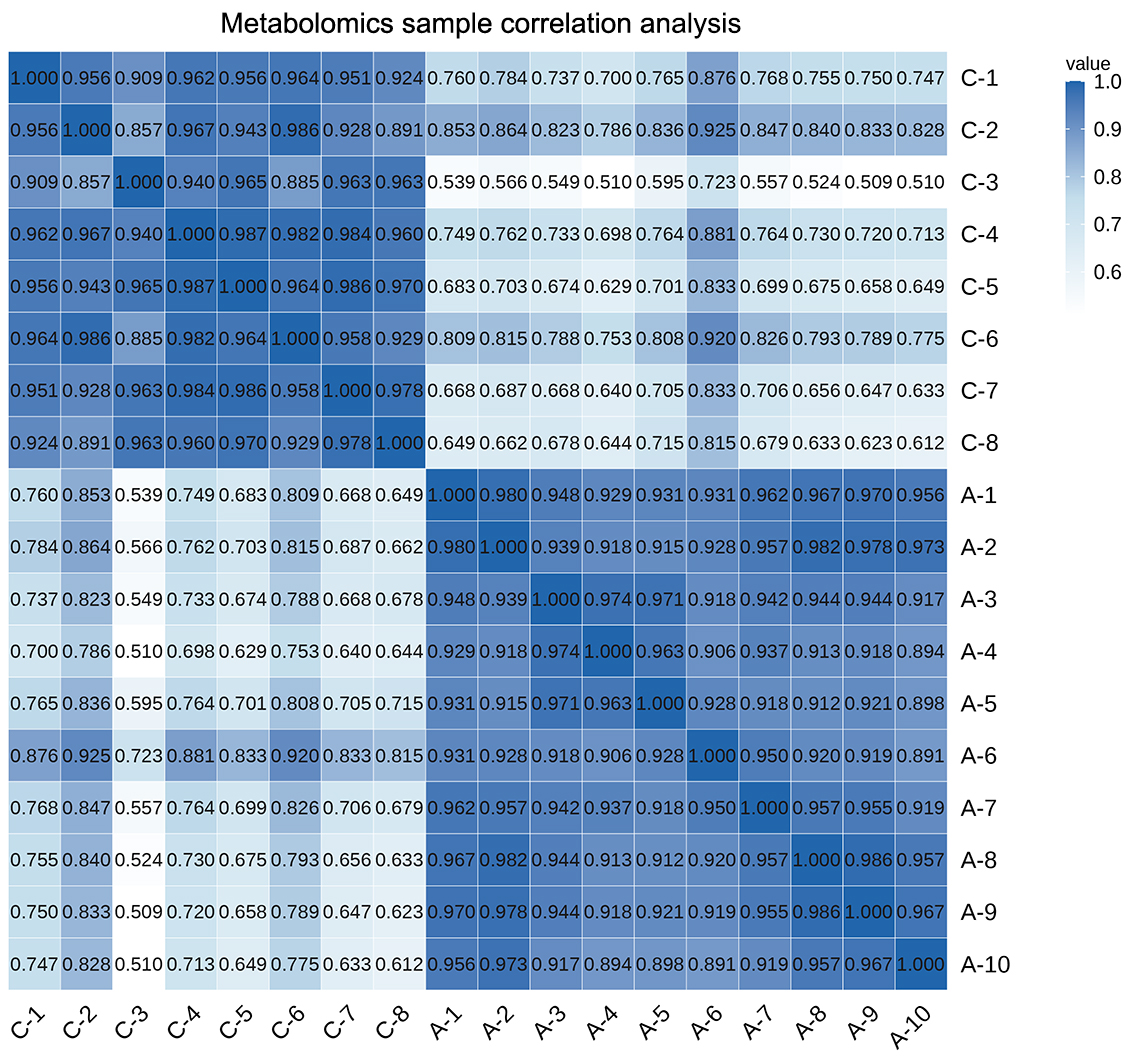
****Figure S1.** Metabolomics sample correlation analysis between the Control (C-1~8) and AVMC (A-1~10) groups. The closer the correlation coefficient is to 1, the higher the similarity in metabolic composition and abundance between samples.

**
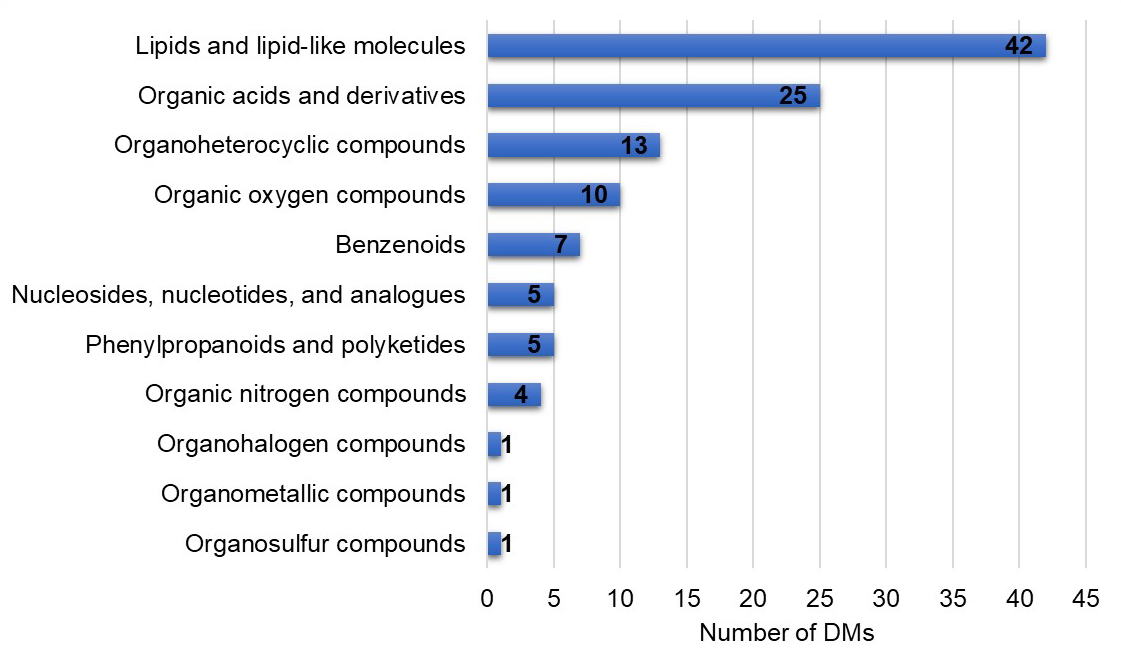
Figure S2.** Superclass classification annotation of the identified DMs according to the HMDB.


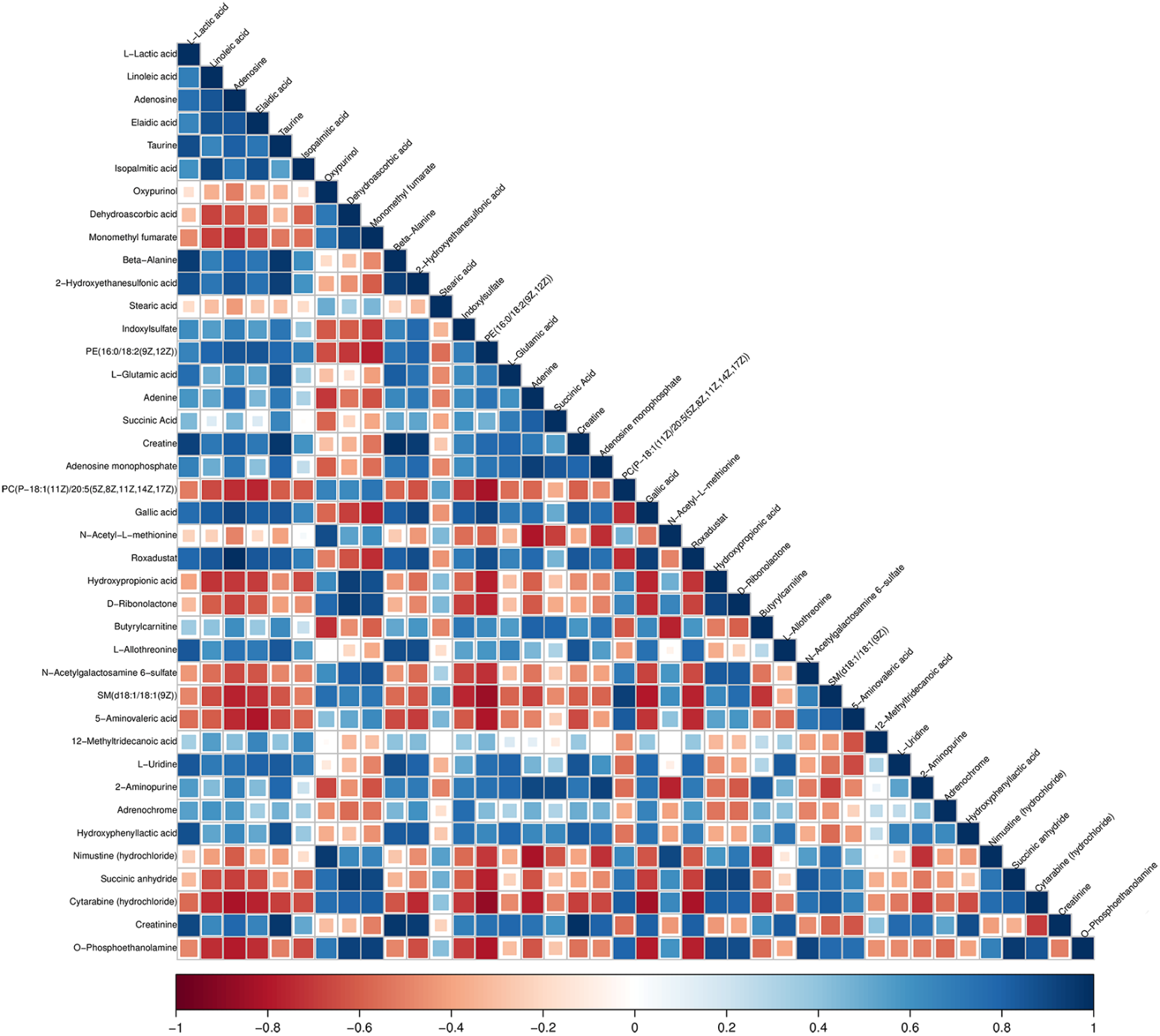
**Figure S3.** Pearson correlation analysis between the top 40 DMs with the most significant VIP values at the MS2 level. Blue color represents positive correlation, and red color represents negative correlation; the deeper the color, the higher the correlation.

**Figure S4.** Proteomics sample correlation analysis between the Control (C-1~8) and AVMC (A-1~10) groups. The closer the correlation coefficient is to 1,
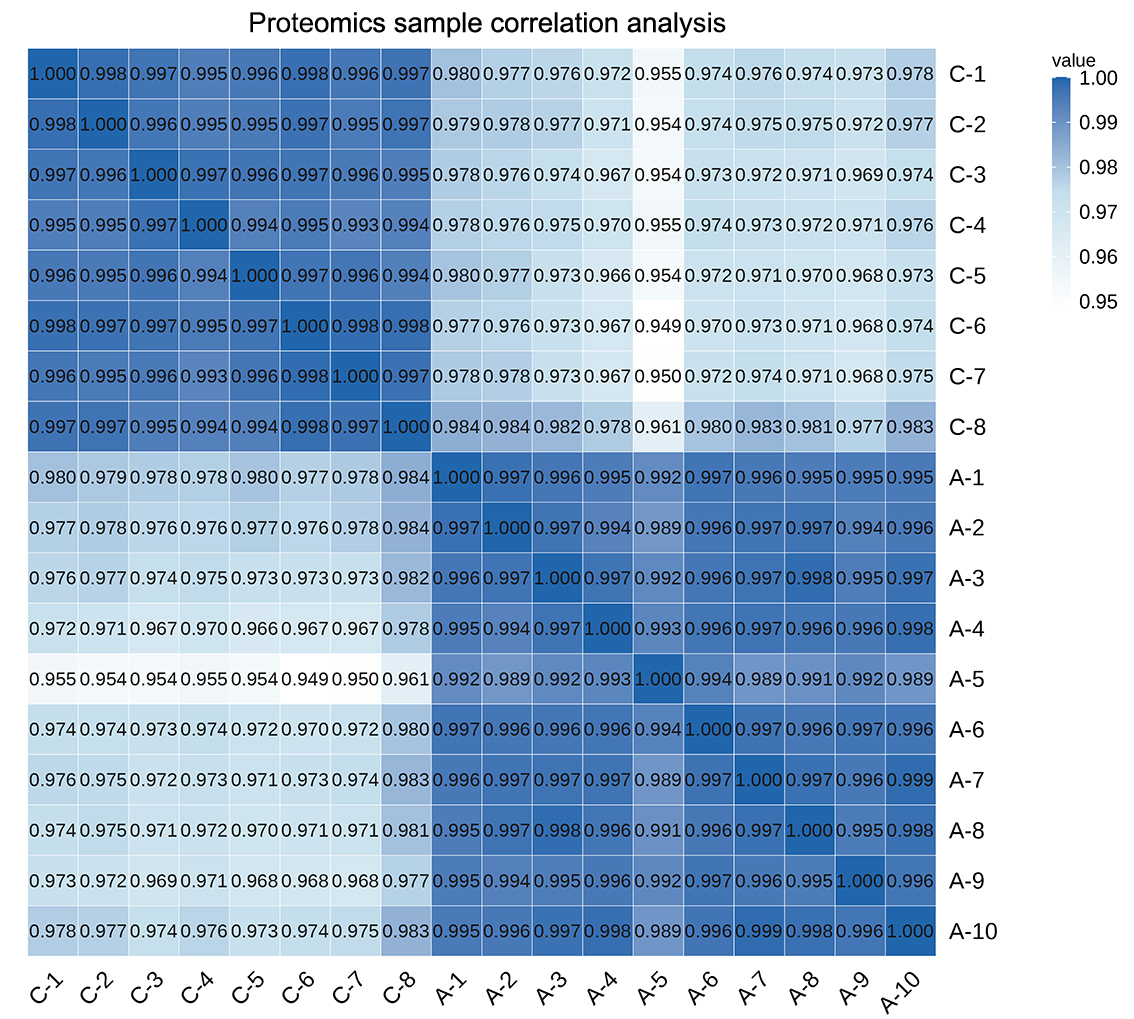
 the higher the similarity of protein expression patterns between samples.
